# Reversible Cerebral Vasoconstriction Syndrome Secondary to Dermatologic Cream: A Case Report

**DOI:** 10.1159/crn/abyag007

**Published:** 2026-08-26

**Authors:** Mark Edward Wilhelm, Andrew Joseph Sessions, Gitanjali Das, Benson H. Sederholm, Jennifer Juhl Majersik

**Affiliations:** a Department of Neurology, School of Medicine, University of Utah, Salt Lake City, UT, USA; b School of Medicine, University of Utah, Salt Lake City, UT, USA; c Department of Pharmacotherapy, College of Pharmacy, University of Utah, Salt Lake City, UT, USA; d Department of Pharmacy Services, University of Utah Health, Salt Lake City, UT, USA

**Keywords:** Reversible cerebral vasoconstriction syndrome, Central nervous system, Imiquimod, Topical medication, Adverse effect, Case report

## Abstract

**Introduction:**

Imiquimod is an FDA-approved prescription topical medication used in the treatment of skin lesions and neoplasms, such as actinic keratosis, superficial basal cell carcinoma, and external genital and perianal warts. The etiology of rare CNS effects, including headache and stroke, is not completely understood. Headache is a commonly reported adverse reaction, while stroke has been identified through postmarketing pharmacovigilance reports without a definitively established causal relationship.

**Case Presentation:**

A 60-year-old female with mild depression and basal cell carcinoma, and an absence of traditional vascular risk factors, presented with a 2-week history of new recurrent thunderclap headaches. CT angiogram showed 40–50% narrowing of the proximal right A1. Conventional angiography demonstrated multiple regions of vasoconstriction which improved with intra-arterial verapamil, supporting the diagnosis of reversible cerebral vasoconstrictive syndrome (RCVS) diagnosis. Oral verapamil was up-titrated to 40 mg every 3 h for vasospasm and headache control. At 4-week follow-up, there was only residual A1 stenosis. Pre-RCVS, the patient had been diagnosed with basal cell carcinoma and was prescribed imiquimod 5% cream, with instructions to apply one 0.25-g prefilled packet topically once daily, which she used for 8.5 weeks prior to symptom onset. The patient had been on a stable low dose (2.5 mg daily) of escitalopram, a selective serotonin reuptake inhibitor and recognized potential trigger of RCVS, for several years without prior neurological symptoms. Discontinuation after the first headache did not prevent subsequent episodes, making it less likely to be the sole precipitant, though a contributory role cannot be excluded. In the absence of other vasoactive substances or identifiable etiologies for vasculitis, we suspect that topical imiquimod was responsible for RCVS in this case.

**Conclusion:**

Imiquimod is an immune modulating agent that acts as a toll-like receptor (TLR) 7 agonist, causing cytokine induction, inflammatory infiltration, and tumor cell apoptosis. Though headache and stroke are reported as rare side effects, stroke subtype and etiology are uncharacterized, with no RCVS reported to our knowledge. A moyamoya animal model, a pathophysiologically distinct entity from RCVS, demonstrated that using imiquimod to induce TLR-7 activation resulted in a phenotypic change in vascular smooth muscle cells. In vivo, imiquimod also led to diminished internal carotid artery blood flow, while inhibiting TLR-7 resulted in decreased stenosis. These findings suggest that imiquimod-mediated TLR-7 agonism can directly alter cerebrovascular smooth muscle behavior – a mechanism that is biologically plausible as a contributor to the acute vasospasm observed in RCVS. Topical imiquimod is thought to trigger systemic reactions secondary to increased production and circulation of cytokines originating from dermal absorption. Further studies are warranted to evaluate the risk of topical imiquimod use as it relates to cerebral vascular emergencies. We recommend that clinicians consider systemic effects of topical medications as potential contributors to neurologic sequelae.

## Introduction

Reversible cerebral vasoconstriction syndrome (RCVS) is a neurovascular disorder characterized by thunderclap headaches that are often recurrent. It is caused by reversible multifocal narrowing of the cerebral arteries and can include neurological deficits related to ischemic or hemorrhagic stroke, seizure, or edema. Cerebral vasoconstriction typically improves within 3 months, and clinical outcome is often benign; however, presentations with associated ischemic stroke, lobar hemorrhage, or subarachnoid hemorrhage can result in death or disability [1]. Ischemia can occur due to reduced perfusion secondary to the stenosis of cerebral arteries [2]. Thunderclap headache may be caused by stimulation of trigeminal afferents located in the leptomeninges, possibly related to early distal arterial involvement or small vessel dysfunction [3].

The etiology of RCVS is multifactorial and may be associated with various conditions including pregnancy, migraine, and medications [4]. While the exact pathophysiological mechanism is not fully known, it is thought that sympathetic nervous system hyperactivity contributes to cerebral vascular tone dysregulation and endothelial dysfunction [5].

Imiquimod is an FDA-approved prescription topical medication used in the treatment of skin lesions and neoplasms, such as actinic keratosis, superficial basal cell carcinoma, and external genital and perianal warts. The etiology of rare CNS effects associated with imiquimod is not completely understood. Headache is a commonly reported adverse reaction in clinical trials of imiquimod, occurring in approximately 5–8% of treated subjects across indications [6]. Stroke has been identified through postmarketing pharmacovigilance reports; however, as these are voluntarily reported from an exposed population of uncertain size, without standard evaluation, a causal relationship to imiquimod has not been definitively established [6]. To our knowledge, RCVS has not been previously reported in association with imiquimod use. Here, we report a case of RCVS suspected to be secondary to use of topical imiquimod cream. This case report was prepared in accordance with the CARE guidelines, and the completed checklist is provided as online supplementary Material (for all online suppl. material, see https://doi.org/10.1159/crn/abyag007).

## Case Presentation

A 60-year-old woman with a past medical history notable for depression and basal cell carcinoma, and an absence of traditional vascular risk factors such as hypertension, diabetes mellitus, hyperlipidemia, or tobacco use, presented after her fourth severe thunderclap headache over the course of 2 weeks. She reported that the initial episode occurred on a rafting trip when she was paddling in cold water. The headaches were described as sudden onset and diffuse, with severe symptoms lasting for minutes to 1 h before improving to a moderate intensity lasting about 6 h before resolution. They initially tended to be frontal or occipital but eventually were severe and holocephalic. Each episode was several days apart from the prior. Some were triggered by rapid head movements and Valsalva maneuvers associated with urination and bowel movements. She denied any associated confusion, fever, visual symptoms, or seizures. The patient had no prior history of thunderclap headaches. No other new vasoactive medications or illicit substances were identified.

Initial laboratory studies were unremarkable, including normal white cell count, erythrocyte sedimentation rate, C-reactive protein and serum drug screen. CT angiogram of the head demonstrated approximately 40–50% focal narrowing of the proximal right A1 segment. MRI brain showed multiple punctate acute cerebral infarcts within the periphery of the bilateral ACA and MCA territories.

Diagnostic digital subtraction cerebral angiogram demonstrated multiple regions of vasoconstriction in the right vertebral artery, right internal carotid artery, left internal carotid artery, and left vertebral artery. Intra-arterial verapamil administration demonstrated responsivity of the intracranial vasculature, supporting the diagnosis of RCVS. Oral verapamil was up-titrated to 40 mg every 3 h for vasospasm and symptomatic headache control, with a gradual taper to every 4, 6, 8, and 12 h at weekly intervals before discontinuation. At 4-week follow-up, repeat CT angiogram showed residual but less conspicuous right A1 stenosis, and other vessels were widely patent without flow-limiting stenosis.

Prior to the patient’s RCVS diagnosis, she was diagnosed with basal cell carcinoma and was prescribed 0.25 g packets of 5% USP imiquimod cream applied topically daily. Imiquimod exposure preceded RCVS onset by approximately 8.5 weeks. The patient had been on a stable low dose (2.5 mg daily) of escitalopram, a selective serotonin reuptake inhibitor and recognized potential trigger of RCVS due to its serotonergic effects on cerebral vascular tone, for several years without recent dose adjustment. Discontinuation of escitalopram after the first thunderclap headache did not prevent subsequent headaches or cerebral vasoconstriction, making it less likely to be the sole precipitant. However, a contributory or synergistic role of chronic selective serotonin reuptake inhibitor use in the setting of imiquimod-induced cytokine activation cannot be excluded. Imiquimod was discontinued and, with concurrent administration of a calcium channel blocker, was associated with improvement in patient’s clinical symptoms over time. No other risk factors were identified.

## Discussion

RCVS is a unifying term to describe a group of disorders characterized by reversible segmental narrowing and dilatation of the cerebral arteries [7]. There are many known risk factors associated with RCVS, including vasoactive medications (often serotonergic medications), illicit drugs (such as marijuana, methamphetamine, or cocaine), and the postpartum state [4]. Idiopathic cases have been reported as well [8]. Patients with RCVS usually present with headache, which is often – but not universally – a sudden, severe thunderclap headache that recurs over several days or weeks. Patients may present with or without focal neurological deficits, and with or without concurrent ischemic or hemorrhagic stroke. The headache may occur spontaneously or be provoked by exercise, as was the case in our patient. Valsalva maneuvers tend to exacerbate the pain.

Clinicians diagnose RCVS based on the recurrent thunderclap headache as well as characteristic brain imaging findings of reversible multifocal segmental narrowing of the cerebral arteries [9]. Digital subtraction angiography is the gold standard investigation [10]. Cerebrospinal fluid is typically normal; however, elevated protein can sometimes be seen [11]. Pathology is only rarely available and shows unremarkable vessels without evidence of arterial inflammation [12].

During local treatment for basal cell carcinoma, our patient developed a classic recurrent thunderclap headache. Our patient may have developed RCVS due to the use of imiquimod, an immune-modulating agent. There are reports of RCVS secondary to steroid and immunosuppressant use, such as cyclophosphamide, tacrolimus, and interferon-α [13]. Imiquimod is a toll-like receptor (TLR) 7 agonist, which causes cytokine induction, inflammatory infiltration, and tumor cell apoptosis [14]. Though headache and stroke are reported as rare side effects, stroke subtype and etiology are uncharacterized, and to our knowledge RCVS has not been previously reported [6]. Measured serum concentrations following topical application of imiquimod are minimal [6]. Differences in surface area application, frequency of administration, dermal thickness and keratinization, heat exposure, or presence of highly inflamed or vascularized areas of skin can contribute to an increase of systemic absorption of topical medications [15]. A correlation between increased serum concentrations of imiquimod and reported systemic reactions is not well characterized from clinical trials. The process by which topical imiquimod triggers systemic reactions is uncertain. It likely results, however, not from systemic imiquimod concentrations, but rather secondary to an increased production and subsequent circulation of cytokines that originated from dermal induction following imiquimod exposure [16].

Moyamoya disease and RCVS are pathophysiologically distinct entities: moyamoya is a chronic, progressive steno-occlusive vasculopathy, whereas RCVS involves acute, transient, and reversible cerebral vasoconstriction. Nevertheless, a moyamoya mouse model demonstrated that TLR-7 activation by imiquimod induced phenotypic changes in vascular smooth muscle cells and diminished internal carotid artery blood flow [17]. These findings suggest that imiquimod-mediated TLR-7 agonism can directly alter cerebrovascular smooth muscle behavior and impair cerebral perfusion – mechanisms that are biologically plausible contributors to the acute vasospasm observed in RCVS. Topical imiquimod likely induces systemic reactions due to the increased production and circulation of cytokines following dermal exposure [18]. Further research is needed to assess the risk of topical imiquimod in relation to RCVS and stroke.

Several recognized RCVS triggers were present in this case and warrant discussion. The patient’s initial thunderclap headache occurred during vigorous physical exertion in cold water, and some subsequent episodes were provoked by Valsalva maneuvers associated with urination and bowel movements. Physical exertion, cold exposure, and Valsalva maneuvers are well-documented precipitants of RCVS. However, these transient physiological stressors are common and do not typically provoke RCVS in isolation. The temporal relationship between imiquimod initiation and symptom onset, together with the biological plausibility of TLR-7-mediated vascular smooth muscle dysfunction, suggests that imiquimod may have predisposed the cerebral vasculature to dysregulation, lowering the threshold at which these common physiological triggers could precipitate vasoconstriction.

In conclusion, this case highlights a potential association between topical imiquimod and RCVS. While recognized RCVS precipitants including physical exertion, cold exposure, and Valsalva maneuvers were present, with a potential contributory role of chronic low-dose escitalopram, we suspect that topical imiquimod may have also contributed to the development of RCVS in this patient. The temporal relationship between imiquimod initiation and symptom onset, combined with the biological plausibility of TLR-7-mediated cerebrovascular dysregulation, supports this hypothesis. Although causality cannot be definitively established from a single case, further pharmacovigilance and mechanistic investigation are needed to clarify whether TLR-7 agonism may contribute to cerebral vascular tone dysregulation. Clinicians managing patients with stroke should thoroughly review all medications for potential contribution to stroke onset, including those administered topically.

## Statement of Ethics

This retrospective review of patient data did not require ethical approval in accordance with local/national guidelines. Written informed consent was obtained from the patient for publication of the details of their medical case.

## Conflict of Interest Statement

The authors have no conflicts of interest to declare.

## Funding Sources

This study was not supported by any sponsor or funder.

## Author Contributions

M.E.W. contributed to conceptualization, data curation, and investigation, and wrote the original draft of the manuscript body; he also participated in manuscript review and editing. A.J.S. contributed to writing the original draft of the abstract and to manuscript review and editing. G.D. contributed to investigation and validation of the clinical diagnosis and participated in manuscript review and editing. B.H.S. contributed to writing the original draft of the abstract and to manuscript review and editing. J.J.M. contributed to conceptualization, supervision of the clinical diagnosis and management, validation, and manuscript review and editing.

## Data Availability

All data generated or analyzed during this study are included in this article and its online supplementary material. Further inquiries can be directed to the corresponding author.
